# Survey and analysis of knowledge, attitude and practice among otolaryngologists in a state in eastern India in relation to the coronavirus disease 2019 pandemic

**DOI:** 10.1017/S0022215120001644

**Published:** 2020-07-29

**Authors:** S Banerjee, S Sarkar, S N Bandyopadhyay

**Affiliations:** 1 Department of ENT and Head Neck Surgery, University Hospitals Coventry and Warwickshire, Coventry, UK; 2 Department of ENT and Head Neck Surgery, All India Institute of Medical Sciences, Bhubaneswar, India,; 3 Department of ENT and Head Neck Surgery, Medical College and Hospital, Kolkata, India

**Keywords:** Surveys And Questionnaires, Coronavirus, Otolaryngology

## Abstract

**Background:**

The coronavirus disease 2019 pandemic has resulted in various changes in knowledge, attitude and practice among doctors. A survey was conducted of otolaryngologists in India regarding these aspects in relation to the coronavirus disease 2019 pandemic.

**Method:**

Otolaryngologists from West Bengal (India) were invited to participate in an online self-administered survey. Data were collected and analysed using appropriate methods.

**Results:**

Responses from 133 participants, grouped into 4 groups by their career stage, were collected and analysed. Of the participants, 36.8 per cent were directly involved in treating a known or suspected coronavirus disease 2019 patient, although 66.2 per cent considered the personal protective equipment inadequate. Ninety-four per cent indicated that their willingness to perform procedures depended on personal protective equipment availability. Of the respondents, 83.5 per cent revealed additional mental stress due to the pandemic. Of the participants, 41.4 per cent took hydroxychloroquine as coronavirus disease 2019 prophylaxis.

**Conclusion:**

This study provides an insight into which issues may need attention, to help ENT surgeons tackle the coronavirus disease 2019 pandemic more effectively based on analysis of responses in the survey.

## Introduction

The World Health Organization (WHO), on 31st December 2019, was informed of pneumonia cases of an unknown origin in Wuhan, China. On 7th January 2020, the micro-organism responsible was identified as a novel coronavirus and temporarily named ‘2019n-CoV’.^1^ On 30th January 2020, it was declared a Public Health Emergency of International Concern in light of its spread. The new coronavirus disease was subsequently named coronavirus disease 2019 (Covid-19) on 11th February 2020, and the outbreak was declared a pandemic by WHO on 11th March, 2020.^2^

The extremely contagious nature of Covid-19 exposes healthcare workers to the risk of infection, with ENT being one of the specialties where healthcare workers are at increased risk of infection. This is a result of exposure to patients’ upper respiratory tracts and the nature of the procedures performed, especially aerosol-generating procedures.^3^ One of the first frontline hospital workers with Covid-19 who passed away in the UK was an otolaryngology consultant.^4^

We conducted a study, using a self-administered anonymous survey, to evaluate the impact of the Covid-19 outbreak on otolaryngologists in India, in order to assess their knowledge, attitude and practice in view of this disease. The state of West Bengal, where this survey study was conducted, has a doctor-to-patient ratio and healthcare infrastructure comparable to the rest of India, and may be considered representative of the nation.

## Materials and methods

ENT and head and neck surgeons, who were members of the Association of Otolaryngologists of India, West Bengal – the state-level chapter of the national professional body of the Association of Otolaryngologists of India – and were living and practising in the state of West Bengal in eastern India, were invited to participate in an online self-administered survey. The survey was designed using Google forms online. The link for participation was distributed through e-mails from the Association of Otolaryngologists of India, West Bengal.

All questions were compulsory unless rendered moot in light of certain answers to previous questions. The questions were designed to assess the knowledge, attitude and practice of the responders. Responses were collected between 18th April 2020 and 26th April 2020. All responses were collected anonymously, summarised, and analysed using appropriate commercially available software (IBM SPSS®, version 1.0.0.1327; Microsoft 365 – Microsoft Corporation).

## Results

There are approximately 350 registered members of the Association of Otolaryngologists of India, West Bengal, out of which 236 members are active and have valid e-mail addresses in our database. Survey questionnaires were not sent out by post to the other members because of feasibility issues. Nineteen members were not presently living in West Bengal and were hence excluded. A total of 133 responses were received in the survey from the 217 e-mails sent, giving a response rate of 61.3 per cent.

The participants were grouped into 4 groups, for ease of analysis, based on their grade on the ENT ladder: group A – trainees or students; group B – post-training experience of less than 5 years; group C – post-training experience of 5–15 years; and group D – post-training experience of more than 15 years. The number of participants in each group were: 22 (16.5 per cent) in group A; 26 (19.5 per cent) in group B; 35 (26.3 per cent) in group C; and 50 (37.6 per cent) in group D. In some instances, where relevant, the participants’ responses were also analysed based on the nature of their primary institute or healthcare setup affiliation.

### Awareness and knowledge data

The source of the first information indicating that the WHO had declared Covid-19 a pandemic was selected as being mass media by large numbers of respondents, with general media (*n* = 69, 52.3 per cent) and the internet (*n* = 47, 35.6 per cent) being top sources. Only six respondents (4.5 per cent) were initially informed by their working institution and five (3.8 per cent) by professional body bulletins (Table 1).

**Table 1.** Source of first information indicating that WHO had declared Covid-19 a pandemic*

Grade of participants	Source of first information					
	Internet	Media	Official statement of working institution	Professional body bulletin or newsletter	Professional colleagues	Total
Trainee or student (group A)	6 (4.5)	12 (9.1)	2 (1.5)	0 (0)	2 (1.5)	22 (16.7)
Post-training experience <5 years (group B)	16 (12.1)	7 (5.3)	0 (0)	1 (0.8)	2 (1.5)	26 (19.7)
Post-training experience 5–15 years (group C)	7 (5.3)	23 (17.4)	3 (2.3)	1 (0.8)	1 (0.8)	35 (26.5)
Post-training experience >15 years (group D)	18 (13.6)	27 (20.5)	1 (0.8)	3 (2.3)	0 (0)	49 (37.1)
Total	47 (35.6)	69 (52.3)	6 (4.5)	5 (3.8)	5 (3.8)	132 (100)

Data represent numbers (and percentages) of responses. Chi-square test *p* = 0.04.

* *n* = 132. WHO = World Health Organization; Covid-19 = coronavirus disease 2019

When asked to self-assess whether their knowledge about Covid-19 was up-to-date and adequate for them as a healthcare professional, the majority were unsure (*n* = 58, 43.6 per cent) and answered ‘maybe’. In contrast, a sizable number (*n* = 29, 21.8 per cent) responded with a definite ‘no’ (Table 2).

**Table 2.** Responses regarding Covid-19 knowledge, practice and PPE*

Response	Consider own knowledge about Covid-19 to be up-to-date & adequate?	Personally been directly involved in treating suspected or confirmed Covid-19 patient?	Feel satisfactorily aware of PPE donning & doffing procedures?
Yes	46 (34.6)	49 (36.8)	99 (74.4)
No	29 (21.8)	84 (63.2)	34 (25.6)
Maybe	58 (43.6)	N/A	N/A

Data represent numbers (and percentages) of responses.

* *n* = 133. Covid-19 = coronavirus disease 2019; PPE = personal protective equipment; N/A = not applicable

**Fig. 1.**
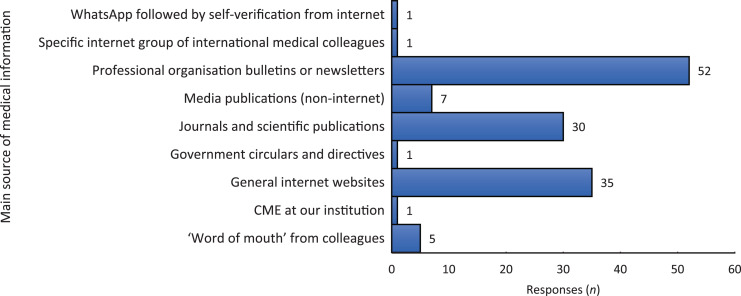
Main source of medical information regarding coronavirus disease 2019. CME = continuing medical education

The main source of medical information regarding Covid-19 was selected as professional body bulletins or newsletters (*n* = 52, 39.1 per cent), followed by general internet websites (*n* = 35, 26.3 per cent), and scientific journals and publications (*n* = 30, 22.6 per cent) (Figure 1).

### Risk assessment data

**Fig. 2.**
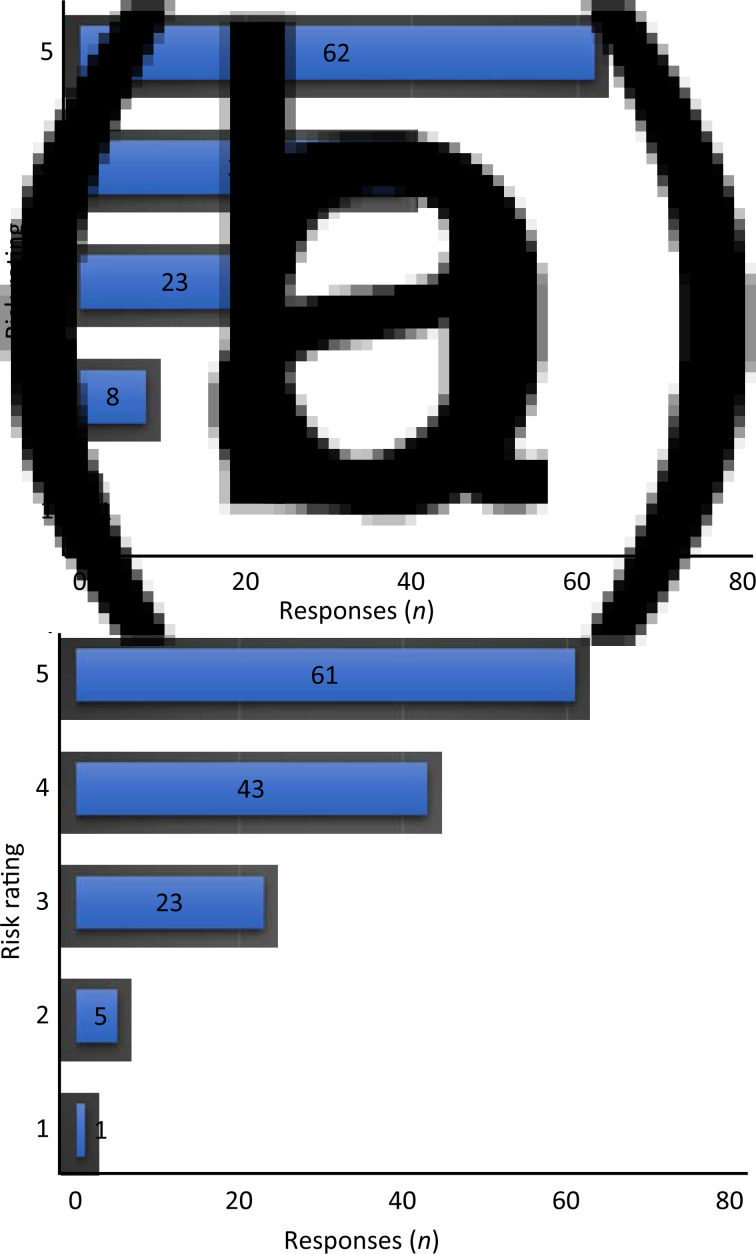
Risk ratings due to coronavirus disease 2019 (Covid-19): (a) risk rating of healthcare facilities being overwhelmed by Covid-19 patients; and (b) risk rating of personal nosocomial infection from working.

Participants rated the risk of their healthcare facilities being overwhelmed by Covid-19 patients on a scale of 1 (extremely unlikely) to 5 (extremely likely). Of the participants, 101 (75.9 per cent) rated the risk as 4 or above, with only 1 participant rating the risk as 1 (Figure 2a). A total of 104 participants (78.2 per cent) rated the risk of a personal nosocomial Covid-19 infection from work as 4 or above, with only 6 participants (4.5 per cent) selecting a risk of 2 or less (Figure 2b). When asked whether ENT surgeons were a higher risk group among healthcare professionals, an overwhelming 131 participants (98.5 per cent) chose a definitive ‘yes’, with the rest opting for ‘maybe’.

### Professional practice data

Participants were asked whether they personally had been directly involved in treating a suspected or confirmed Covid-19 patient to date. Group A (trainees and students) was the only group in which more than half of the participants responded with ‘yes’ (12 out of 22 participants, 54.5 per cent). The difference among groups was statistically significant (chi-square test *p* = 0.04).

Most of the participants who had personally been involved in treating a suspected or confirmed Covid-19 patient were found to be attached to a medical college (government or private) (*n* = 30) or hospital only (*n* = 18). This finding was also statistically significant (chi-square test *p* < 0.001) (Table 3).

**Table 3.** Practice and PPE responses according to participants’ affiliation type

Participants’ affiliation type	Personally been directly involved in treating suspected or confirmed Covid-19 patient?			Consider sufficient PPE of adequate quality is available?			
	Yes	No	Chi-square test *p*-value	Yes	No	Prefer not to answer	Chi-square test *p*-value
Medical college (government or private) (*n* = 66)	30 (45.5)	36 (54.5)	<0.001	4 (6.1)	50 (75.8)	12 (18.2)	0.002
Hospital only (government or private) (*n* = 39)	18 (46.2)	21 (53.8)		6 (15.4)	21 (53.8)	12 (30.8)	
Hospital, private chamber (*n* = 1)	1 (100)	0 (0)		1 (100)	0 (0)	0 (0)	
Nursing home (*n* = 6)	0 (0)	6 (100)		2 (33.3)	2 (33.3)	2 (33.3)	
Private chamber (*n* = 20)	0 (0)	20		0 (0)	15 (75.0)	5 (25.0)	
Private practice with major attachment at private hospital (*n* = 1)	0 (0)	1 (100)		1 (100)	0 (0)	0 (0)	
Total (*n* = 133)	49 (36.8)	84 (63.2)		14 (10.5)	88 (66.2)	31 (23.3)	

Data represent numbers (and percentages) of responses, unless indicated otherwise. PPE = personal protective equipment; Covid-19 = coronavirus disease 2019

An overwhelming 118 participants (88.7 per cent) revealed they would be unwilling to perform a non-emergency procedure on a known or suspected Covid-19 patient. However, the majority (87.3 per cent) indicated that they would consider operating in emergency cases. There were no statistically significant differences between the responses among the career grade groups for either question (chi-square test *p* = 0.94 for non-emergency procedures, and *p* = 0.18 for emergency procedures). A vast majority of the participants (*n* = 125, 94 per cent) felt that their answers to the above questions would depend on the availability of suitable personal protective equipment (PPE) (Table 4).

**Table 4.** Willingness to perform procedures*

Response	Willing to perform non-emergency procedure on suspected or confirmed Covid-19 patient?	Willing to perform emergency procedure on suspected or confirmed Covid-19 patient?	Willingness dependent on PPE availability?
Yes	8 (6.0)	61 (45.9)	125 (94.0)
No	118 (88.7)	17 (12.8)	8 (6.0)
Maybe	7 (5.3)	55 (41.4)	N/A

Data represent numbers (and percentages) of responses.

* *n* = 133. Covid-19 = coronavirus disease 2019; PPE = personal protective equipment; N/A = not applicable

**Fig. 3.**
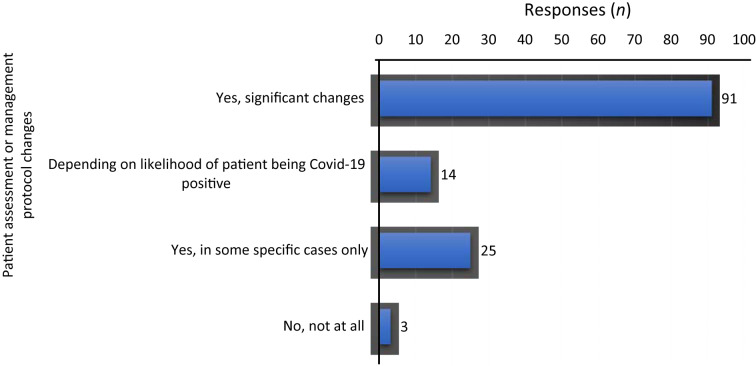
Changes made (or not) in patient assessment or management protocols as a result of coronavirus disease 2019 (Covid-19) outbreak.

Most participants (*n* = 91, 68.4 per cent) had made changes in their protocols because of Covid-19; only three respondents (2.3 per cent) denied making any changes whatsoever. Some stated that they had made changes only in specific cases (*n* = 25, 18.8 per cent), while others (*n* = 14, 10.5 per cent) said that changes made were solely based on the likelihood of the patients being Covid-19 positive (Figure 3).

Those who reported making changes to clinical protocols (130 out of 133 participants, 97.8 per cent), stated that these involved amendments to clinical examination protocols (*n* = 120, 92.3 per cent) and decision-making protocols for planned procedures (*n* = 100, 76.9 per cent). Only 26 respondents (20 per cent) indicated that they had altered their drug prescription protocols.

### Personal protective equipment data

Eighty-eight participants (66.2 per cent) felt there was insufficient good quality PPE at work, while 31 respondents (23.3 per cent) selected the option ‘prefer not to answer’. The difference in responses across the career grade groups was not statistically significant (chi-square test *p* = 0.1). However, when the responses of the participants were grouped according to their primary healthcare affiliation, the difference was statistically significant (chi-square test *p* = 0.002) (Table 3). Only 4 out of 66 participants (6.1 per cent) from medical colleges (government and private) and 6 out of 39 participants (15.4 per cent) from only hospitals said they had sufficient PPE of adequate quality in their healthcare setting for their usage.

Most respondents (*n* = 85, 63.9 per cent) stated that they had bought or wished to buy PPE personally for their protection. The percentage increased across the groups with increasing experience or grade, ranging from 11 participants (50 per cent) in group A (trainees and students) to 38 participants (76 per cent) in group D (more than 15 years’ post-training experience). However, there was no statistically significant difference (chi-square test *p* = 0.10) in responses between the groups.

Thirty-four participants (25.6 per cent) felt that they were not satisfactorily aware of the PPE donning and doffing procedures (Table 2). Of those who considered themselves adequately aware of these procedures (*n* = 99), the source of such knowledge was mostly from online webinars of professional bodies (*n* = 60, 60.6 per cent), followed by general videos on the internet (*n* = 34, 34.3 per cent). Only 27 respondents (27.2 per cent) declared that they had received formal training at their own (*n* = 23, 23.2 per cent) or another (*n* = 4, 4 per cent) healthcare institution.

### Mental health data

Of the surgeons, 83.5 per cent (111 of 133) thought that Covid-19 had caused high levels of mental stress in their professional life (4 or above, on a scale of 1 to 5). The groups on the extreme ends of the ENT grade ladder had a higher percentage of participants selecting 5 as a response (group A – 13 out of 22 participants, 59.1 per cent; group D – 29 out of 50 participants, 58 per cent).

The participants ranked their specific reasons for additional mental stress as per their level of impact, with 1 = ‘most important’, 5 = ‘least important’ and ‘not present’ = no impact. The reasons provided for ranking mental stress were ‘financial changes’, ‘risk of personal infection and suffering’, ‘risk of infecting family member’, ‘social stigma for working with Covid-19 patients’ and ‘worries about family support in case of death’.

**Fig. 4.**
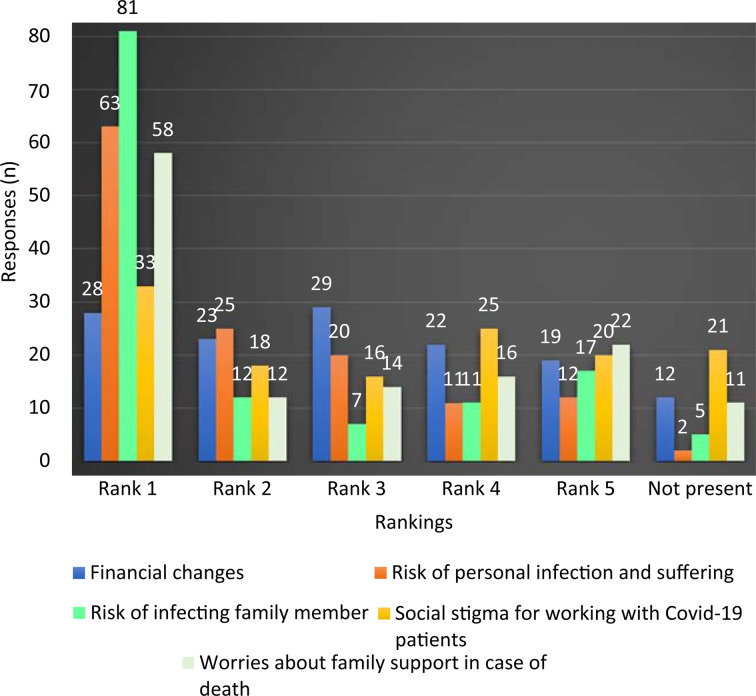
Ranking of reasons for additional mental stress related to coronavirus disease 2019 (Covid-19).

Friedman's test was used to determine mean rank. The mean rank values were: 2.67 for ‘risk of infecting family member’, 2.87 for ‘risk of personal infection and suffering’, 2.98 for ‘worries about family support in case of death’, 3.13 for ‘social stigma for working with Covid-19 patients’ and 3.36 for ‘financial changes’. The result was statistically significant, with a *p*-value of 0.001 (Figure 4).

### Prophylaxis data

The Indian Council of Medical Research issued a recommendation on 22 March 2020 for the empirical use of hydroxychloroquine as a prophylactic agent against severe acute respiratory syndrome coronavirus-2 (SARS-CoV-2) infection.^5^ We asked the participants whether they had taken hydroxychloroquine as prophylaxis against Covid-19.

**Fig. 5.**
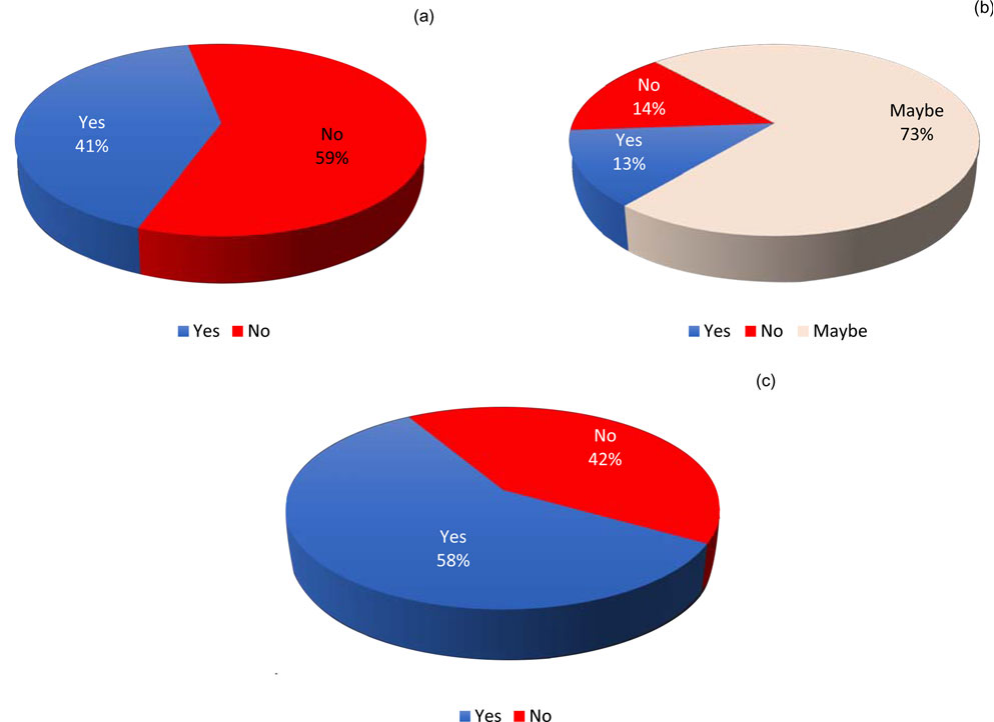
Responses to the following questions regarding using hydroxychloroquine as prophylaxis against coronavirus disease 2019 (Covid-19): (a) did you take hydroxychloroquine as prophylaxis?; (b) are you confident that hydroxychloroquine has prophylactic benefit in Covid-19?; and (c) were you confident about the prophylactic dosage of hydroxychloroquine?

Seventy-eight participants (58.7 per cent) denied taking hydroxychloroquine as prophylaxis, including 19 out of 22 participants (86.4 per cent) in career grade group A. The highest number of hydroxychloroquine takers for prophylaxis was in group C (22 out of 35 participants, 62.9 per cent) followed by group B (11 out of 26 participants, 42.3 per cent). The difference across the groups was statistically significant (chi-square test *p* = 0.003) (Figure 5a).

Only 7 of the 55 participants who took hydroxychloroquine prophylaxis (12.7 per cent) were confident that hydroxychloroquine would be effective in Covid-19 prevention. A further 40 participants (72.7 per cent) answered ‘maybe’, while the remaining 8 (14.6 per cent) answered ‘no’ (Figure 5b).

Twenty-three out of the 55 participants (41.8 per cent) stated that they were not confident about the dosage of hydroxychloroquine to take, even though the official Indian Council of Medical Research statement had defined a specific regimen for the medicine (Figure 5c).

## Discussion

### Awareness and knowledge

Interestingly, most surgeons found out about Covid-19 from the media (52.3 per cent) or internet (35.6 per cent), rather than professional bodies. The small percentage of respondents informed by their working institution (4.5 per cent) and professional body bulletins (3.8 per cent) may be due to a lag of essential news propagation by these channels or because people are less dependent on these sources for obtaining urgent information.

Of the respondents, 39.1 per cent selected professional body bulletins or newsletters as their main source of medical information regarding Covid-19. This emphasises the proactive role that professional bodies play globally in disseminating the rapidly changing guidelines and medical information regarding Covid-19. It reiterates the fact that although healthcare professionals often obtain news from sources such as Twitter, when it comes to acquiring knowledge about a medical problem, the majority look to professional bodies for guidelines.

### Risk assessment

Of respondents, 75.9 per cent estimated that the risk of their healthcare facilities being overwhelmed by Covid-19 patients was high (4 or above, on a scale of 1 to 5), and 78.2 per cent felt there was a high risk of personal infection.

There were no statistically significant differences between responses when grouped according to career grade (Kruskal–Wallis test *p* = 0.55) or type of institutional affiliation (Kruskal–Wallis test *p* = 0.80). This implies that the perceived risk of healthcare facilities being overwhelmed by Covid-19 patients was similar across all categories of ENT surgeons.

Of the participants, 98.5 per cent considered ENT surgeons as being in a higher risk group among healthcare professionals. The notable point here is that, irrespective of the years of experience in practice, not a single participant denied the increased risk as an ENT surgeon. Similar results were noted in a survey for ophthalmology practitioners, wherein 80 per cent of respondents felt they were at a high risk of Covid-19 transmission while working.^6^

### Professional practice

The least experienced clinicians were the only group with a majority having treated suspected or confirmed Covid-19 cases, which could mean that the surgeons in training were more exposed at the frontline than the experienced surgeons. While the fatality rate of Covid-19 presumably increases with age, it is sure to raise the ethical question of whether the surgeons lower in the hierarchy are doing this service of their own volition. It is also noteworthy that among the least experienced clinicians, who had the most frontline workers in their group, the majority confessed that their knowledge was inadequate. Conversely, the most experienced surgeons, who were least involved in direct Covid-19 patient care, were comparatively more confident of their knowledge of the disease. This disparity might be because the group that is handling patients feel their knowledge is not sufficient to face the practical problems which the other group is not facing.

The majority of surgeons who were personally directly involved in treating suspected or confirmed Covid-19 patients were attached to medical colleges (government or private) and hospitals. This reflects that these types of institutions were bearing the brunt of exposure to Covid-19 patients, as expected.

Of the participants, 88.7 per cent were unwilling to perform a non-emergency procedure on a known or suspected Covid-19 patient. This is in line with internationally accepted guidelines advising the avoidance of non-emergency work to a maximum extent. However, 94 per cent of the participants also stated that their willingness to perform procedures would depend on the availability of suitable PPE, which is understandable.

Of the participants, 68.4 per cent had made changes in their protocols of patient assessment and management. Of the respondents, 18.8 per cent said their amendments would be implemented only in special cases if the patient is suspected of having Covid-19 symptoms. However, this is something to ponder given the possibility of asymptomatic carriers.

Only 20 per cent of the participants changed their prescription habits. However, given that certain guidelines initially recommended against the usage of drugs such as ibuprofen in Covid-19 patients, advice which has subsequently been altered, it is prudent to keep oneself updated by reliable sources regarding prescription protocols.^7^

### Personal protective equipment

The debates regarding adequate PPE, both in terms of quality and quantity, have been raging globally since the start of the pandemic. This debate is likely to continue given the contagious nature of the disease and the risk of exposure for contacts.

Of the participants, 66.2 per cent overall, and 90.9 per cent from among the least experienced clinicians, stated that they did not have sufficient PPE of adequate quality at work. It was concerning that 31 respondents (23.3 per cent) selected ‘prefer not to answer’, which highlights the apprehension among the medical fraternity globally that raising concerns about PPE availability and quality may land them in trouble with the authorities concerned. This apprehension is not desirable in any part of the world. Only 6.1 per cent of participants from medical colleges and 15.4 per cent from other hospitals stated that they had adequate quality PPE in appropriate quantities. This is a matter of concern, considering that medical colleges and hospitals are supposed to be referral centres which bear the brunt of maximum exposure to Covid-19 cases.

Of the participants, 63.9 per cent stated that they had bought or wished to buy PPE personally for their protection. The percentage increased across the groups with more experience. This could reflect a feeling of increased vulnerability of the older surgeons to the ill-effects of Covid-19 infection, or the affordability of the kits.

Of the participants, 25.6 per cent felt that they were not satisfactorily aware of the PPE donning and doffing procedures. Of those who thought they were satisfactorily aware of the procedures, 27.2 per cent stated that they had received formal training at their own (23.2 per cent) or another (4 per cent) healthcare institution. This highlights the importance of scientific webinars and communications from professional bodies, and the need to step up formal training at healthcare institutions, which should receive prime importance. This also highlights the lack of initiative in certain educational institutes to offer training to healthcare personnel. This issue should be taken seriously. Indeed, a literature search revealed a protective effect of real-time training on infection prevention and control measures (odds ratio = 0.12, *p* = 0.0072).^8^

### Mental health

Of the surgeons, 83.5 per cent selected 4 or above (on a scale of 1 to 5) when asked to rate the additional mental stress in their professional life due to Covid-19. This shows the considerable stress that ENT surgeons are facing in their professional lives because of this pandemic, and is probably in line with the global healthcare fraternity if not the whole world.

When asked about the reasons for the additional mental stress, most participants were primarily worried about infecting their family and/or themselves, and the family support required if they become infected and die, which is in line with the Indian social structure.

### Prophylaxis

Of the trainee or student participants, 86.4 per cent denied taking hydroxychloroquine as prophylaxis as per the Indian Council of Medical Research recommendations. This was an interesting finding, as their group had the highest percentage of participants directly involved in treating confirmed or suspected Covid-19 patients. Only 38 per cent of the most senior participants took hydroxychloroquine as prophylaxis, which could be because of the higher risk of hydroxychloroquine side effects in people with cardiac co-morbidities in a presumably higher age group, or the reluctance to try something empirical until further evidence is available.

Only 12.7 per cent of those participants who took hydroxychloroquine as prophylaxis were confident that hydroxychloroquine has a prophylactic benefit in Covid-19; a further 72.7 per cent were not sure and answered ‘maybe’. However, this did not prevent them from taking hydroxychloroquine as prophylaxis, reflecting a sense of desperation to try anything for prevention.

- The coronavirus disease 2019 (Covid-19) pandemic has affected healthcare professionals all around the world
- A few survey studies have investigated effects on doctors, but most are related to personal protective equipment use
- This survey study details and analyses knowledge, attitude and practice among otolaryngologists from eastern India regarding the Covid-19 pandemic
- The study investigated effects of the pandemic from the perspective of otolaryngologists in a developing nation

Of the participants who took hydroxychloroquine as prophylaxis, 41.8 per cent stated that they were not confident about the dosage of hydroxychloroquine. Hydroxychloroquine is considered to be a relatively safe drug, and is used widely in India as an antimalarial drug and in the treatment of autoimmune disorders. However, the Indian Council of Medical Research had reinforced that its usage should not instil a false sense of security, especially as the wrong dosage could lead to side effects or failure in prophylaxis.^5^

## Conclusion

The effects of the global Covid-19 pandemic have been unprecedented, and the subsequent consequences are expected to last for a considerable time. This paper provides an insight into Covid-19 related practice and experience among otolaryngologists in West Bengal. The state of West Bengal, being one of the highly affected states in India, and having a healthcare setup that is comparable with most parts of India, could be deemed representative of the nation in terms of tackling Covid-19.

## References

[1] World Health Organization. Coronavirus disease (COVID-19) pandemic. In: http://www.euro.who.int/en/health-topics/health-emergencies/coronavirus-covid-19/novel-coronavirus-2019-ncov [19 April 2020]

[2] World Health Organization. WHO Director-General's opening remarks at the media briefing on COVID-19–11 March 2020. In: https://www.who.int/dg/speeches/detail/who-director-general-s-opening-remarks-at-the-media-briefing-on-covid-19---11-march-2020 [19 April 2020]

[3] ENTUK. COVID-19 Letter to Members 1. In: https://www.entuk.org/covid-19-letter-members-1 [19 April 2020]

[4] The Guardian. NHS consultant dies from Covid-19. In: https://www.theguardian.com/world/2020/mar/29/first-nhs-consultant-dies-from-covid-19#maincontent [19 April 2020]

[5] ICMR. National Task Force for Covid 19. Recommendation for empiric use of hydroxy-chloroquine for prophylaxis of SARS-CoV-2 infection. In: https://icmr.nic.in/sites/default/files/upload_documents/HCQ_Recommendation_22March_final_MM_V2.pdf [26 April 2020]

[6] Minocha A, Sim S, Than J, Vakros G. Survey of ophthalmology practitioners in A&E on current COVID-19 guidance at three major UK eye hospitals. Eye 2020;34:1243–5 32265510 10.1038/s41433-020-0857-5 PMC7136748

[7] Gov.UK. Government response: Commission on Human Medicines advice on ibuprofen and coronavirus (COVID-19). In: https://www.gov.uk/government/news/commission-on-human-medicines-advice-on-ibuprofen-and-coronavirus-covid-19 [26 April 2020]

[8] Guo X, Wang J, Hu D, Wu L, Gu L, Wang Y Survey of COVID-19 disease among orthopaedic surgeons in Wuhan, People's Republic of China. J Bone Joint Surg Am 2020;102:847–54 32271208 10.2106/JBJS.20.00417 PMC7188039

